# Factors Regulating the Potential for Freshwater Mineral Soil Wetlands to Function as Natural Climate Solutions

**DOI:** 10.1007/s13157-024-01893-6

**Published:** 2025-01-08

**Authors:** Shizhou Ma, Purbasha Mistry, Pascal Badiou, Sheel Bansal, Irena F. Creed

**Affiliations:** 1 School of Environment and Sustainability, University of Saskatchewan, Saskatoon, SK Canada; 2 Ducks Unlimited Canada, Stonewall, MB Canada; 3 Northern Prairie Wildlife Research Center, U.S. Geological Survey, Jamestown, ND USA; 4 Department of Physical and Environmental Sciences, University of Toronto Scarborough, Toronto, ON Canada

**Keywords:** Wetland carbon cycle, Carbon sequestration, Greenhouse gas fluxes, Carbon dioxide, Methane, Wetlandscape

## Abstract

There are increasing global efforts and initiatives aiming to tackle climate change and mitigate its impacts via natural climate solutions (NCS). Wetlands have been considered effective NCS given their capacity to sequester and retain atmospheric carbon dioxide (CO_2_) while also providing a myriad of other ecosystem functions that can assist in mitigating the impacts of climate change. However, wetlands have a dual impact on climate, influencing the atmospheric concentrations of both CO_2_ and methane (CH_4_). The cooling effect associated with wetland CO_2_ sequestration can be counterbalanced by the warming effect caused by CH_4_ emissions from wetlands. The relative ability of wetlands to sequester CO_2_ versus emit CH_4_ is dependent on a suite of interacting physical, chemical, and biological factors, making it difficult to determine if/which wetlands are considered important NCS. The fact that wetlands are embedded in landscapes with surface and subsurface hydrological connections to other wetlands (i.e., wetlandscapes) that flow over and through geochemically active soils and sediments adds a new layer of complexity and poses further challenges to understanding wetland carbon sequestration and greenhouse gas fluxes at large spatial scales. Our review demonstrates how additional scientific advances are required to understand the driving mechanisms associated with wetland carbon cycling under different environmental conditions. It is vital to understand wetland functionality at both wetland and wetlandscape scales to effectively implement wetlands as NCS to maximize ecological, social, and economic benefits.

## Introduction

Climate change is leading to irreversible and devastating consequences to nature and society (Abbass et al. 2022). The rapid increase of greenhouse gases (GHGs) in the atmosphere is the main driver of climate change (Riahi et al. 2011). The Paris Agreement committed to limiting the increase in the Earth’s average temperature to well below 2 °C above pre-industrial levels, while striving to restrict the rise to 1.5 °C. The latest report from the Intergovernmental Panel on Climate Change (IPCC) highlights that achieving net-zero global greenhouse gas (GHG) emissions by 2050 is the essential pathway to keep the rise to Paris Agreement targets (IPCC 2023). Countries (e.g., USA and Canada), in response, have emphasized the potential of implementing natural climate solutions (NCS), which involves protecting, restoring, and improving management of forests, wetlands, and grasslands to help stabilize if not reduce GHGs in the atmosphere (Griscom et al. 2017; Drever et al. 2021).

The concept NCS is often confused with terms nature-based solutions (NbS) and nature-based climate solutions (NbCS), delaying critical climate mitigation actions (Ellis et al. 2024). While NbS encompasses a wider range of actions aimed at addressing various societal challenges (Ferreira et al. 2023), NCS primarily focuses on climate mitigation through net zero carbon dioxide (CO_2_) equivalent GHG emissions (Griscom et al. 2017; Drever et al. 2021). Despite the difference between NCS and NbS, effective wetland NCS actions are implemented in an ecologically and socio-economically responsible manner (Griscom et al. 2017; Ellis et al. 2024), making them capable of delivering numerous ecosystem services (e.g., hydrological regulation, water purification, and biodiversity enhancement) that are essential for human well-being. The concept of NbCS is similar to NCS, but it involves additional activities in modified ecosystems (e.g., macroalgae farming) that have been altered from their original state (Ellis et al. 2024). In contrast, NCS leverage nature’s pre-exiting infrastructure of biological, physical, and chemical mechanisms to mitigate atmospheric GHGs (Griscom et al. 2017; Ellis et al. 2024).

Wetlands are gaining popularity as NCS because of their capacity to remove CO_2_ from the atmosphere. More specifically, wetlands can actively sequester atmospheric CO_2_ through plant productivity and subsequent accumulation of plant biomass as detritus in soil organic matter (SOM) (Kayranli et al. 2010; Bernal and Mitsch 2012; Bansal et al. 2023a). Extensive research has demonstrated that wetland conservation (i.e., avoided wetland conversion) is a cost-effective approach with immediate climate benefits, aligning with the Paris Agreement’s timeline (Drever et al. 2021; Schuster et al. 2024; Ma et al. 2024). Yet, the environmental conditions that promote the long-term storage of carbon are also the conditions that promote methane (CH_4_) production and emission, making wetlands collectively a significant global source of CH_4_ (Bridgham et al. 2006). CH_4_ is a more potent GHG, with a much higher radiative efficiency compared to CO_2_ (Neubauer and Megonigal 2015; Cain et al. 2019; Lynch et al. 2020). Therefore, the cooling effect associated with wetland CO_2_ uptake may be offset by the climate warming effect associated with wetland CH_4_ emissions (Neubauer and Megonigal 2015). Recent studies have suggested that if effective wetland management strategies are not implemented, rewetting of drained wetlands can increase CH_4_ emissions, limiting the ability of rewetted wetlands to deliver short-term climate solutions (Schuster et al. 2024; Ma et al. 2024). While policy targets have been implemented to incentivize wetland protection, conservation, and restoration (Drever et al. 2021; Valach et al. 2021), the scientific evidence in support of wetland climate benefits is inconsistent amongst studies. This is largely due to uncertainties associated with the driving mechanisms of wetland GHG flux rates (Bridgham et al. 2006; Ausseil et al. 2015; Nahlik and Fennessy 2016; Loder and Finkelstein 2020), as well as the techniques and models used to measure and quantify wetland GHG flux rates (Liu et al. 2020b).

Wetlands can be classified into peatlands (wetlands where there is water on or near the surface and where there is a buildup of partially decomposed organic matter) and mineral soil wetlands (wetlands where there is water on or near the surface and the soil consists of non-peat accumulating organic soil) (Bridgham et al. 2006; Ausseil et al. 2015). Autochthonous organic matter production under aerobic conditions and accumulation under anaerobic conditions are the main pathways for carbon accumulation in peatlands that are characterized by relatively high carbon content (e.g., > 65%; Ausseil et al. 2015). Meanwhile, allochthonous dissolved and particulate organic matter from upstream catchments as well as autochthonous organic matter production under anaerobic environmental conditions are considered the main sources for carbon accumulation in mineral soil wetlands (Bridgham et al. 2006; Loder and Finkelstein 2020). Resulting from allochthonous dissolved material inputs, nutrient levels and plant productivity are usually high in mineral soil wetlands (Ausseil et al. 2015). Yet, mineral soil wetlands store less organic matter compared to peatlands since they are characterized by faster decomposition rate (Loder and Finkelstein 2020). Most mineral soil wetlands comprise mineral soil substrates, but some comprise > 50% organic soil substrates (Bridgham et al. 2006).

Freshwater mineral soil wetlands are present in numerous large wetland complexes worldwide, such as the Prairie Pothole Region of North America (Bansal et al. 2023b), the Sanjiang Plain of Northeast China (Zhang et al. 2008), and the Florida Everglades (Mitsch and Gosselink 2015). Despite wetlands typically having less carbon stored in their soils as compared to peatlands, they play important roles in regional carbon storage due to their extensive areal extent (e.g., freshwater mineral soil wetlands comprise 95% of the total wetland area in the USA) (Nahlik and Fennessy 2016). However, there is high uncertainty in the carbon sequestration potential and CO_2_ and CH_4_ flux mechanisms of wetlands and therefore their potential as NCS (Bridgham et al. 2006; Ausseil et al. 2015; Nahlik and Fennessy 2016; Loder and Finkelstein 2020; Villa et al. 2020; Creed et al. 2022).

**Fig. 1:**
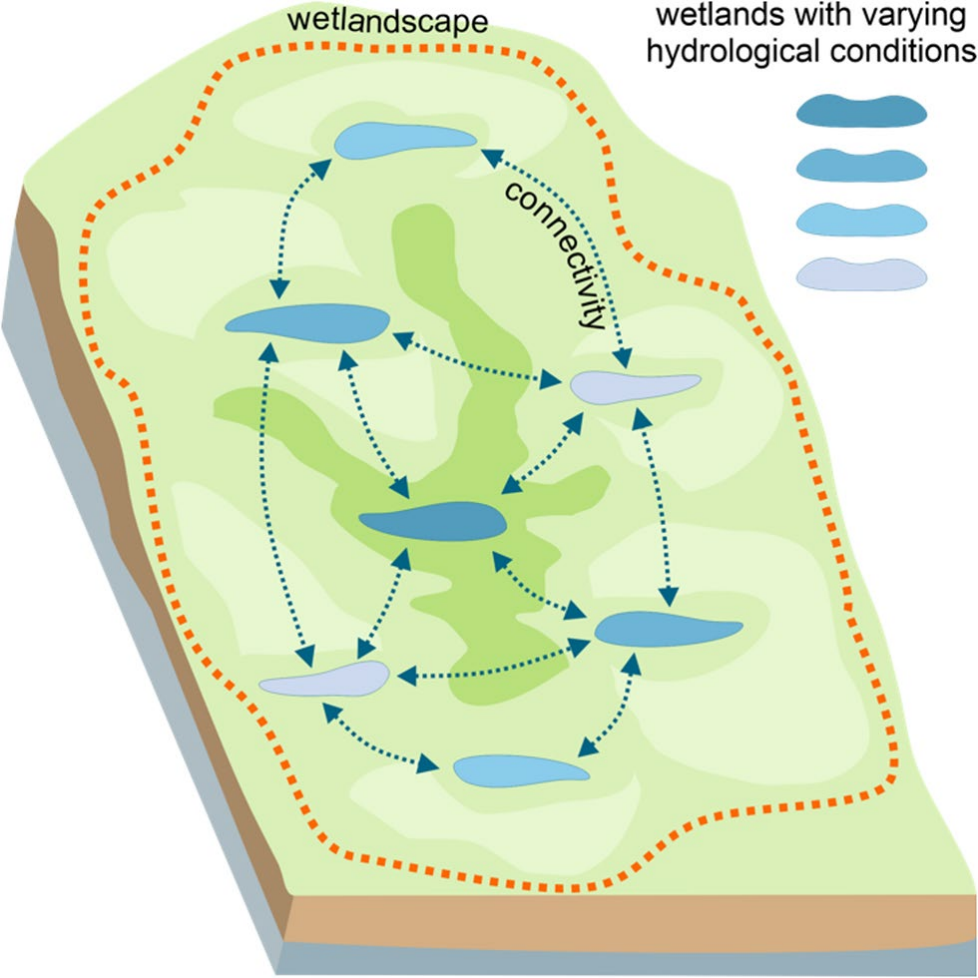
Schematic map illustrating wetland connectivity within a wetlandscape context. Blue color gradient shows individual wetlands with varying hydrological conditions, orange dash line shows the extent of wetlandscape, green color gradient depicts the undulating topography surrounding wetlands

Hydrologically connected wetlands within an associated hydrological catchment are called wetlandscapes (Fig. 1) (Thorslund et al. 2017). Some studies have emphasized the need to switch from a focus on individual wetlands to wetlandscapes when assessing the potential of wetlands as NCS (Thorslund et al. 2017; Åhlén et al. 2020; Hambäck et al. 2023). However, the quantification of the hydrological connectivity of individual wetlands within wetlandscapes remains a major scientific challenge (Golden et al. 2017; Ameli and Creed 2017), and the impact of this hydrological connectivity on wetland carbon sequestration and GHG fluxes remains ambiguous. Despite some progress towards understanding surface and subsurface hydrological flow patterns within wetlandscapes (Ameli and Creed 2019a, b), studies exploring how wetland number, size, organization, and connectivity affect the biogeochemical functions of wetlandscapes remain limited (Cheng and Basu 2017; Hambäck et al. 2023). While carbon fluxes can be restricted to the scale of individual wetlands based on their physical properties, understanding carbon cycling at the scale of wetlandscapes requires explicit consideration of hydrological flow patterns, the biology and chemistry of soils and waters along the hydrological flow path, and the position of wetlands within the wetlandscape (Åhlén et al. 2020).

With increasing local, national, and global interest in wetlands as NCS, the scientific and policy communities need an improved and shared understanding of the GHG-related functions of wetlands (and wetlandscapes) in order to effectively incorporate wetlands as NCS in climate change policies and management strategies. This review paper contributes to this initiative by answering (1) What constitutes a freshwater mineral soil wetland?; (2) How are wetland carbon sequestration and carbon-based GHG fluxes (i.e., CO_2_ and CH_4_) (collectively referred as wetland carbon fluxes) influenced by physical, chemical, and biological factors of freshwater mineral soil wetlands?; (3) How do the number, size, organization, and connectivity of wetlands influence landscape scale wetlands carbon sequestration and carbon-based GHG fluxes?; and (4) are there evidence-based management actions for freshwater mineral soil wetlands as NCS?

## What is a Wetland?

### Wetland Definition

A coherent and readily interpretable definition of a ‘wetland’ could reduce challenges in the implementation of policies related to wetlands as NCS. There are many perspectives on how wetlands are defined reflecting differences in disciplines, contexts, and regulatory frameworks (Table 1) (Cowardin 1979; Scott and Jones 1995; Mitsch and Gosselink 2015; Tiner 2016). Yet, most times these wetland definitions give a vague idea of wetland boundaries given the wide range of variation in wetland hydrology and vegetation in different wetland classes, which adds to the uncertainty of identifying wetland boundaries. Additionally, wetland definitions adopted by different jurisdictions make it difficult to implement a consistent wetland delineation process. For example, the mean depth of wetlands is considered < 2 m in Canada, while it is < 2.5 m in the USA (National Wetlands Working Group 1997; Federal Geographic Data Committee 2013). Richardson et al. (2022) proposed evidence-based definitions that distinguish different inland waterbodies (i.e., ponds, wetlands, and lakes) using surface area, depth, and emergent vegetation cover. Based on Richardson et al. (2022), wetlands are small waterbodies “with no defined surface area”, depth < 1 m, and emergent vegetation coverage > 30%. Inconsistencies in characterizing wetland boundaries create challenges in terms of mapping, monitoring, and modelling wetland carbon fluxes, leading to high uncertainty in regional and national GHG budgets (Melton et al. 2013).

**Table 1:** The definition of freshwater mineral soils wetland under different classification systems

Classification	Definition	Focus of the classification system	Classes	References
The Canadian Wetland Classification System (Canada)	“A wetland is defined as: land that is saturated with water long enough to promote wetland or aquatic processes as indicated by poorly drained soils, hydrophytic vegetation and various kinds of biological activity which are adapted to a wet environment.”	Wetland’s distinct ecological processes	Bog, fen, marsh, swamp, and shallow water	National Wetlands Working Group (1988) and (1997)
Cowardin’s classification system (USA)	“Wetlands are lands transitional between terrestrial and aquatic systems where the water table is usually at or near the surface or the land is covered by shallow water.”	Wetland’s hydrology, soils, and vegetation community composition	Five main systems are Riverine, Lacustrine, Palustrine, Marine, and Estuarine. These are subdivided into subsystems and then classes	Cowardin (1979)
Brinson’s Hydrogeomorphic classification system (USA)	Wetland are “those areas that are inundated or saturated at a frequency to support, and which normally do support, plants adapted to saturated and/or inundated conditions.”	Wetland’s hydrogeomorphic processes that shape the ecosystems	Depressional, riverine, mineral flats, organic flats, tidal fringe, lacustrine fringe, slopes	Brinson (1993)
Stewart and Kantrud classification system (USA and Canada)	This classification is developed for wetlands in prairie pothole region based on the vegetation community structure and distribution which is reflective of water permanence.	Wetland’s vegetation community composition and distribution	Class I - ephemeral ponds, Class II - temporary ponds, Class III - seasonal ponds and lakes, Class IV - semi-permanent ponds and lakes, Class V - permanent ponds and lakes, Class VI - alkali ponds and lakes, and Class VII - fen ponds	Stewart and Kantrud (1971)
Ramsar classification system (International)	Article 1.1: “Wetlands are areas of marsh, fen, peatland or water, whether natural or artificial, permanent or temporary, with water that is static or flowing, fresh, brackish or salt, including areas of marine water the depth of which at low tide does not exceed six meters.” Article 2.1: “may incorporate riparian and coastal zones adjacent to the wetlands, and islands or bodies of marine water deeper than six meters at low tide lying within the wetlands.”	Wetland’s geographic setting	Marine/coastal wetlands, inland wetlands, and human-made wetlands	Matthews (1993), Scott and Jones (1995), Ramsar Information Bureau (1998)

Wetlands are ecosystems with saturated or inundated soils that are submerged for enough time to develop anaerobic conditions that support uniquely adapted vegetation and microbial communities (Mitsch and Gosselink 2015; Poulter et al. 2021; USEPA 2021). Hydrological soil indicators such as soil color analysis are practical tools to distinguish between upland (non-hydrological) and wetland (hydrological) soils and to determine wetland boundaries in the absence of or along with vegetation indicators of the wetland (Schmidt and Ahn 2021).

### Wetland Zonation

**Fig. 2:**
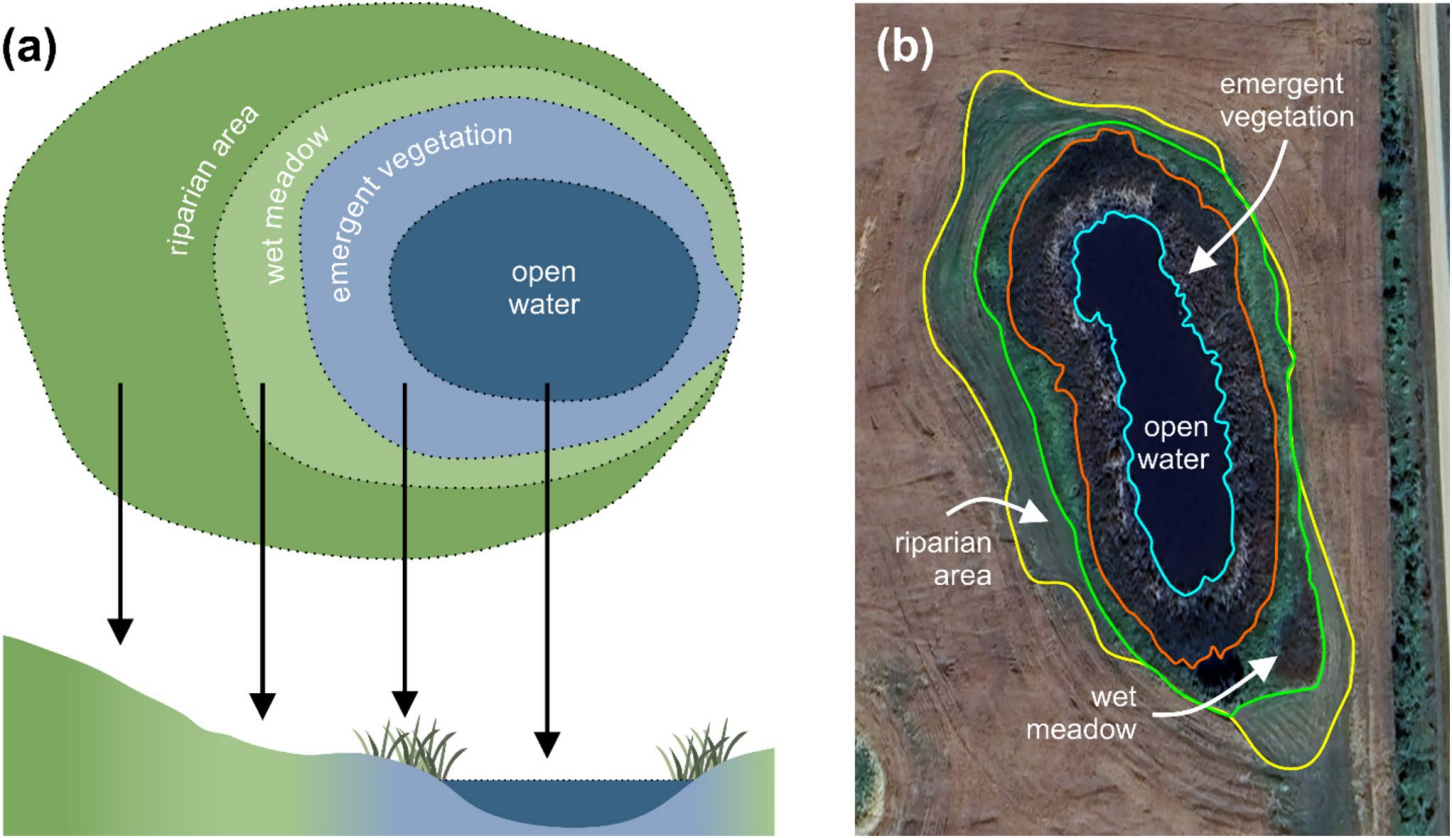
Wetland zonations: (**a**) cartoon showing wetland zonations. (**b**) aerial photograph illustrating wetland zonations on a landscape: open water, emergent vegetation, wet meadow, and riparian zone. Image source: Google Earth Pro (version 7.3), image date August 2023

Wetland area can be discretized into open-water, emergent vegetation, wet meadow, and riparian zones (Fig. 2) (Stewart and Kantrud 1971). The open-water zone can be referred to as the permanent water zone of the wetland, which varies in size depending on the inundation duration, frequency, and intensity (Stewart and Kantrud 1971). The emergent vegetation and wet meadow zones refer to the areas where herbaceous plants grow. For the emergent vegetation zone, plants grow in soils that are submerged for most of the year, whereas in the wet meadow zone the plants grow in soils that are only inundated for shorter periods (e.g., < 1 month) of time. Riparian zones are the interface between the wet meadow zone and upland areas, which are generally covered by native vegetation adapted to the high soil moisture near the edges of wetland (Kattelmann and Embury 1996). Meanwhile, riparian buffers (or buffer strips) are considered portions of the uplands that are characterized by native vegetation compared to managed vegetation on the uplands. The presence of a higher diversity of plant species (adapted to high and low soil moisture) and the lower soil moisture compared to other zones of the wetland hinder the riparian buffer from being recognized as the outer boundary of a wetland according to many definitions (Tiner 2016). Nevertheless, it is widely accepted that the riparian buffer plays an important role in terms of preventing upland nutrients from entering wetland ecosystems and enhancing wetland carbon sequestration potential (Udawatta and Jose 2012; Cole et al. 2020). All zones of the classification system are not necessarily present in all wetlands and the zones frequently shift in position from year-to-year depending on weather (Stewart and Kantrud 1971). Conversely, the actual wetland boundary is relatively stable, and it does not change from year-to-year even if wetland plants or water are not present.

## Wetland Carbon Cycle

The wetland carbon cycle involves processes representing the carbon movement into and out of the ecosystem including carbon uptake, storage, GHG emissions, and lateral inputs/outputs, as well as transformation during decomposition (Reddy and Delaune 2008; Bansal et al. 2023a). Wetlands have five major carbon pools, including plant biomass, microbial biomass, particulate organic carbon (POC), dissolved organic carbon (DOC), and carbon-based gaseous products (e.g., CO_2_ and CH_4_) (Kayranli et al. 2010; Bansal et al. 2023a). The plant biomass carbon pool contains wetland vegetation (e.g., emergent and submergent macrophytes and mosses) and various algal assemblages (phytoplankton and benthic algae) that contribute to the fixation of inorganic carbon (e.g., CO_2_ and/or bicarbonate [HCO_3_^−^]) to organic carbon (Kayranli et al. 2010). Several studies have shown that aboveground biomass (AGB) (e.g., leaf and stem biomass) can be substantial source of soil organic carbon (SOC) pool during growing season (Bernal and Mitsch 2013; Pan et al. 2024). Yet, much of it is either rapidly decomposed or exported from the wetland system (Mitsch and Gosselink 2015; Pan et al. 2024). Belowground biomass (BGB) (e.g. root biomass and fungi), on the other hand, is often incorporated directly into the soil profile and inherently protected by root carbon within soil aggregates (Osland et al. 2018; Pan et al. 2024). Despite vary by wetland types, research indicates that BGB contributions generally can exceed those from AGB by a factor of two or more, often accounting for the majority (e.g., > 60%) of carbon stored in wetland soils over the long term (Pan et al. 2024). Microbes represent a small portion of the soil carbon pool, yet they are crucial for soil organic matter decomposition, transforming organic carbon back to inorganic forms and mineralizing POC and DOC. Microbial biomass usually contributes < 5% of total carbon in wetland soil and water (USEPA 2008). POC and DOC contribute to the total organic carbon in surface waters, with POC contributing to the major organic carbon pool (i.e., ~ 95% of the organic carbon pool is above the soil surface) (Davidson and Janssens 2006; Reddy and Delaune 2008). During decomposition processes, chemical constituents of plant detritus (e.g., lignin and cellulose) are decomposed by microbes produced extracellular enzymes, resulting in the formation of POC, DOC, and carbon-based gaseous end products (Gessner et al. 2010). The dead bodies of organism (e.g., benthic invertebrates, amphibians, and fish) that reside in wetlands further favour the bacterial decomposition processes (Bansal et al. 2023a; Hu et al. 2023). DOC refers to soluble forms of organic carbon that pass through a glass fiber filter with a pore size range from 0.2 to 0.7 μm (Creed et al. 2018). Despite accounting for a relatively small portion of the total organic carbon in soil (< 1%), DOC represents a large portion (~ 90%) of the total organic carbon in surface water and runoff (Reddy and Delaune 2008; Kayranli et al. 2010).

CO_2_ and CH_4_ are gaseous, inorganic, end products formed through decomposition of organic matter. They can be released either as dissolved inorganic carbon (DIC) via surface runoff or as gaseous forms into the atmosphere. CO_2_ is produced under both aerobic and anaerobic conditions, while CH_4_ is produced under anaerobic conditions by methanogens using CO_2_ as an electron acceptor (Poffenbarger et al. 2011; Herbert et al. 2015). To be more specific, under anaerobic conditions, complex organic matter is decomposed through various processes, including fermentation and methanogenesis, which are biological processes governed by the soil microbial community, carbon substrate supply, and the presence of alternative electron acceptors (Morrissey et al. 2014a). Fermentation converts complex organic matter to various organic acids, alcohols, and gases (e.g., CO_2_) (Reddy and Delaune 2008; Morrissey et al. 2014a), leading to the subsequent production of CH_4_ through methanogenesis facilitated by a specific group of Archaea known as methanogens (Mitsch and Gosselink 2015; Neubauer and Megonigal 2021). Apart from fermentation, CO_2_ can be produced when sulfate reduction occurs in wetlands with abundant salts or sulfate ions (Pester et al. 2012; Mitsch and Gosselink 2015). Under aerobic conditions, CO_2_ is produced by autotrophic respiration. In wetland systems, aerobic respiration is more effective in terms of breaking down organic matter as compared to anaerobic processes (e.g., fermentation and methanogenesis). Therefore, CO_2_ almost always accounts for most wetland GHG emissions on a mass basis (Kayranli et al. 2010; Neubauer and Megonigal 2021).

**Fig. 3:**
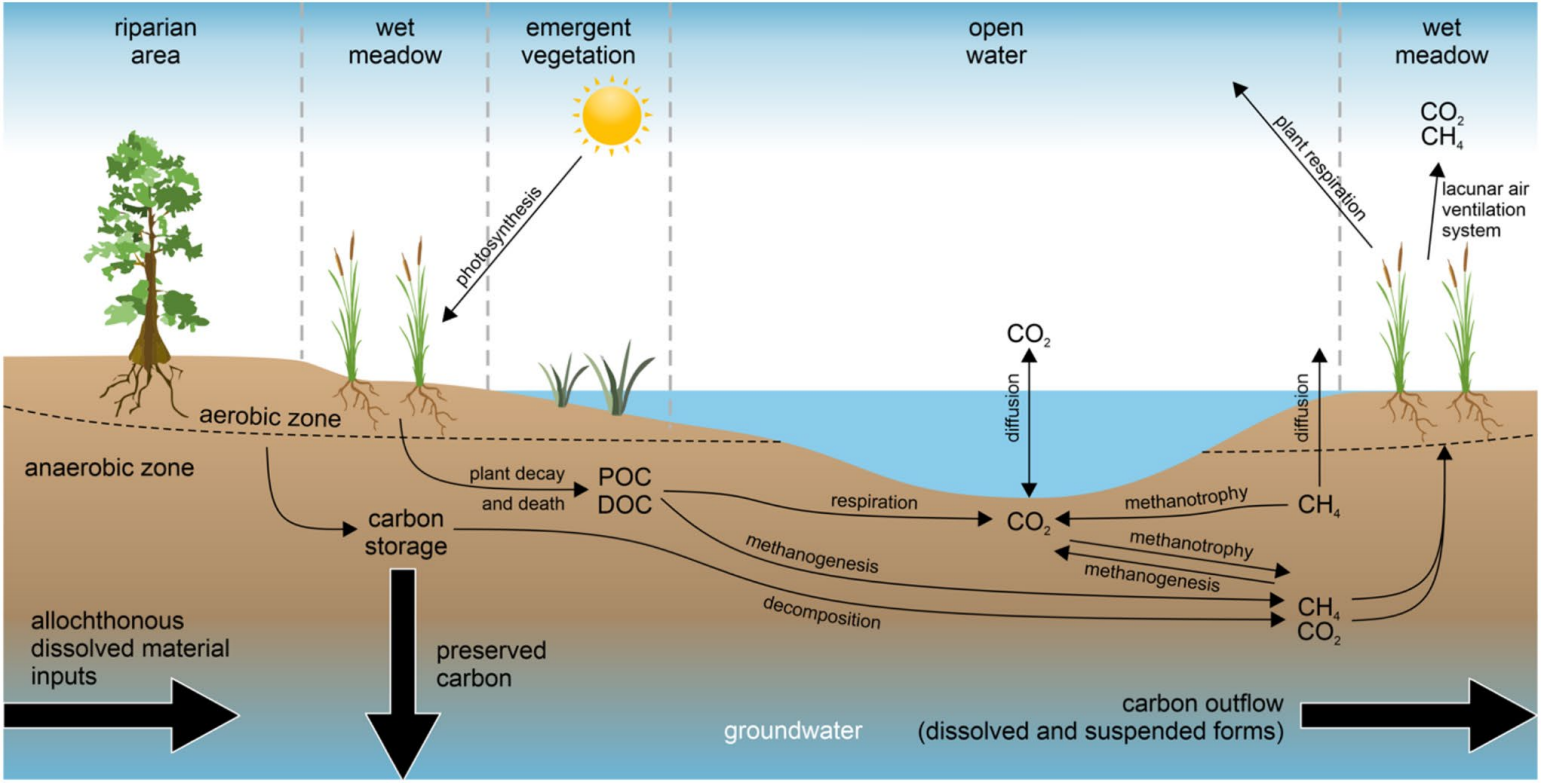
Schematic map depicting wetland profile and carbon turnover processes

The wetland carbon cycle can be described as organic (i.e., POC and DOC) and inorganic (i.e., DIC, CO_2_, and CH_4_) pools and the fluxes among these pools (Fig. 3) (Davidson and Janssens 2006; Bansal et al. 2023a). Carbon inputs to wetlands can be classified as autochthonous and allochthonous inputs. Autochthonous carbon inputs are mainly comprised of organic carbon from decomposing wetland detrital biomass as well as CO_2_ fixation by wetland plants and microbes (i.e., algae). Given the ability of algae to assimilate atmospheric CO_2_ and transform it into organic carbon compounds through photosynthesis, algae-derived organic matter are considered important autochthonous carbon sources in wetland ecosystem (Krause-Jensen et al. 2018). This is particularly true for eutrophic wetlands that experience severe algal blooms that produce labile organic matter. When the blooms senescence results in anaerobic conditions, much of this organic matter is converted to CH_4_ (Krause-Jensen et al. 2018). Allochthonous carbon inputs, on the other hand, are in particulate and dissolved forms from flowing waters and runoff (Hupp et al. 2019). Various physio-chemical (e.g., hydrolysis and redox reactions) and biological processes (e.g., fermentation and methanogenesis) are involved in the breakdown of input carbon, which consequently results in carbon exported as particulate or dissolved forms to adjacent surface and groundwater systems or emitted back into the atmosphere as gaseous end products (i.e., CO_2_ and CH_4_) (Pant et al. 2003; Neubauer and Megonigal 2021). Given the waterlogged and often anaerobic conditions in wetlands, anaerobic decomposition predominates, which is far less efficient in the breaking down of carbon as compared to carbon uptake via photosynthesis (Villa and Bernal 2018). The imbalance between primary productivity and decomposition under anaerobic conditions results in organic matter accumulating in wetland sediments (Davidson and Janssens 2006; Kayranli et al. 2010).

## Physical, Chemical, and Biological Controls on Wetland Carbon Cycle

**Fig. 4:**
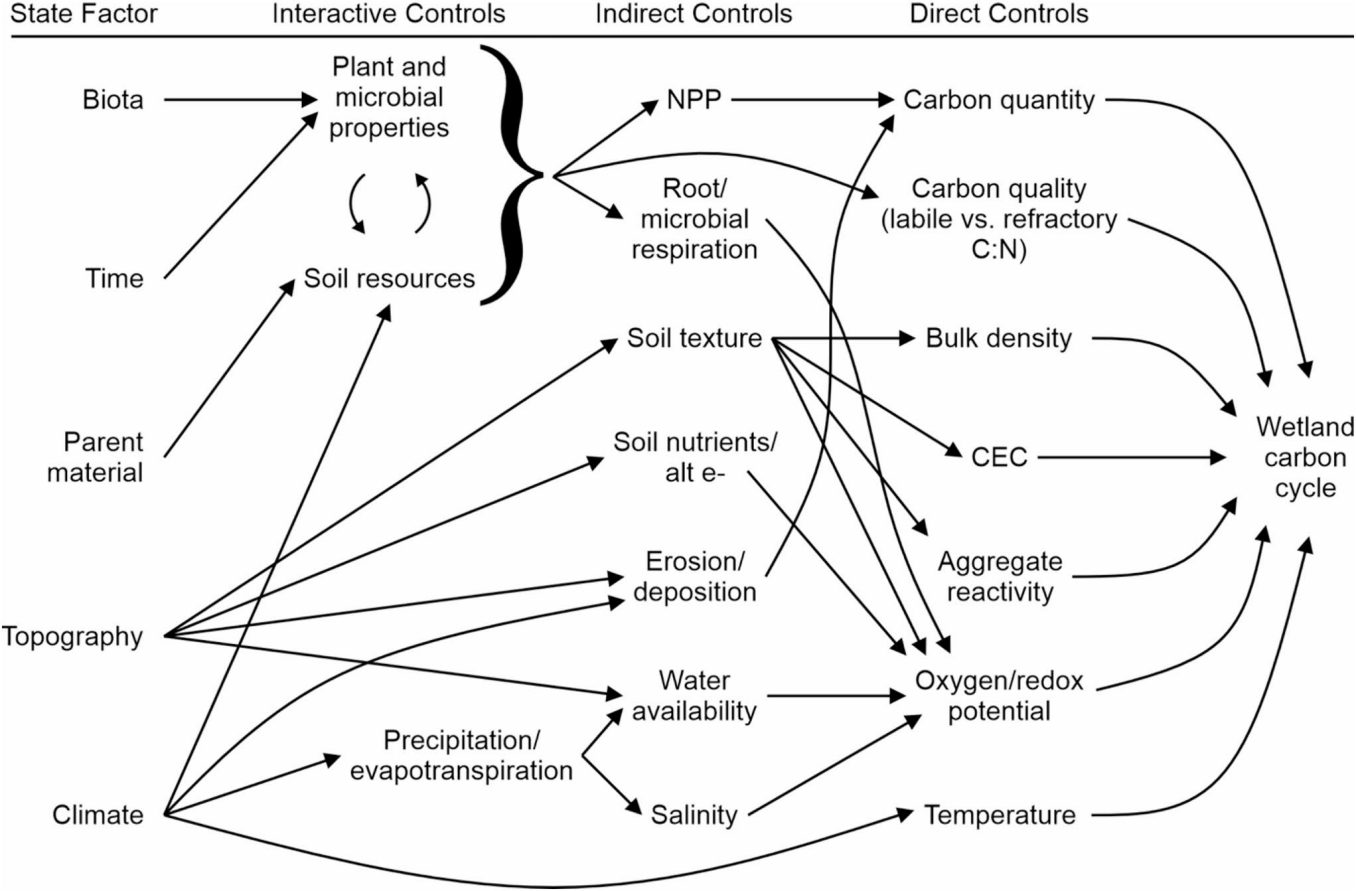
Conceptual diagram showing the influence of indirect and direct factors on wetland carbon cycling processes, including carbon sequestration and greenhouse gas (GHG) flux rates. C: N stands for carbon to nitrogen ratio; CEC stands for cation exchange capacity; NPP stands for net primary productivity; alt e- stands for alternative terminal electron acceptors

Wetland carbon cycling rates depend heavily on myriad factors that interactively, indirectly, and directly regulate wetland physical, chemical, and biological conditions (Fig. 4). Given the inherent complexity of wetland ecosystems, different controlling factors are not independent of each other, regulating the overall dynamics of wetland carbon cycling. For instance, a shift in wetland biota characteristics, such as plant community composition, influences net primary productivity, which regulates carbon substrate quantity and ultimately organic carbon sequestration rate (Kayranli et al. 2010; Villa and Bernal 2018). Here, we introduce how five state factors—biota, time, parent material, topography, and climate (after Jenny 1941 and Chapin et al. 2011)—influence wetland carbon cycling. The following sub-sections describe how various physical, chemical, and biological controls under each state factor interactively, indirectly, and directly influence the rates of carbon cycling in wetlands.

### Biota and Time

#### Plant Community

Living wetland plant biomass captures atmospheric CO_2_ during photosynthesis, producing simple sugars that are later synthesized into complex compounds like lignin and cellulose, which contribute to the formation of leaves, stems, and roots. As plants age, portions of the older aboveground and belowground plant biomass senesces, detaches, and becomes sources of autochthonous carbon to the wetland (Gessner et al. 2010; Were et al. 2019). Studies have shown that different wetland plant types are characterized by distinct primary production capacity, resulting in different wetland carbon cycling rates (Davila and Bohlen 2021). For instance, Lishawa et al. (2010) found that wetlands with non-native emergent aquatic macrophytes like *Typha angustifolia* and *T.* × *glauca* (collectively *Typha spp*.) have larger and deeper stocks of soil organic matter compared to wetlands dominated by native vegetation. Bansal et al. (2019) highlighted the impact of *Typha spp.* on wetland carbon cycling, suggesting that the high net primary productivity rate of *Typha spp.* results in a relatively large carbon stock in *Typha spp.* dominated wetlands.

While plants sequester CO_2_, they also affect GHG effluxes. For example, emergent and submergent aquatic macrophytes control CO_2_ and CH_4_ production and emission processes by serving as providers of carbon substrates (Kayranli et al. 2010; Bansal et al. 2020; Ueyama et al. 2023). Labile organic matter that is recently senesced and plant root exudates can trigger a priming effect, causing a rapid increase of organic matter decomposition (Kuzyakov 2010; Guenet et al. 2018; Theus et al. 2023). While high carbon inputs mean more carbon can be potentially sequestered in the soils, it also results in greater sources of carbon substrates to be mineralized and exported back to the atmosphere as GHGs or to downstream environments in dissolved and particulate forms (Table 2) (Villa and Bernal 2018).

**Table 2:** The influence of direct controls on carbon sequestration and greenhouse gas (GHG) emissions. The + sign indicates a positive relationship; the higher the magnitude of the direct controls, the higher the rate of carbon sequestration or GHG emissions. The – sign indicates a negative relationship; the higher the magnitude of the direct controls, the lower the rate of carbon sequestration or GHG emissions. The +/- sign indicates complex relationships between the direct controls and the carbon sequestration or GHG emissions. No Info indicates that to the best of our knowledge no direct relationship exists or the relationship remains unexplored in the literature

Name of the direct controls	Influence on carbon sequestration	Influence on CH_4_ emission	Influence on CO_2_ emission	References
Carbon quantity	+	+	+	Pant et al. (2003), Mazurczyk and Brooks (2018), Li et al. (2019)
Carbon quality (C: N)	-	+	+	Kuzyakov (2010), Guenet et al. (2018), Davila and Bohlen (2021), Krull et al. (2001), Mazurczyk and Brooks (2018), Tang et al. (2018), Luo et al. (2019), Hu et al. (2024), Yi et al. (2023)
Bulk density	-	-	+	Horn and Smucker (2005), Brown et al. (2017), Tangen and Bansal (2020)
Cation exchange capacity (CEC)	+	No info	-	Krull et al. (2001), Vogel et al. (2014), Dong et al. (2022)
Soil aggregate reactivity	-	+	+	Luo et al. (2017), Brown et al. (2017), Wang et al. (2019a, b)
Redox potential	-	-	+	Baldwin et al. (2006), Herbert et al. (2015), Kayranli et al. (2010), Lacroix et al. (2019)
Temperature	+/-	+	+/-	Bridgham et al. (2006), Davidson and Janssens (2006), Conant et al. (2011), Pugh et al. (2018), Bansal et al. (2023b)

The lacunar air ventilation system of specific wetland plant type can serve as pathways for CH_4_ passing through the sediment-water-atmosphere continuum. For instance, the lacunar air ventilation system of *Typha* spp. provides a route for CH_4_ to be rapidly emitted directly from wetlands to the atmosphere, bypassing the zones of oxidation and slow diffusion process in the water column (Bansal et al. 2020). In fact, the presence of *Typha* spp. can account for > 50% of CH_4_ emissions from wetlands (Rose and Crumpton 2006; Moseman-Valtierra 2012; McInerney and Helton 2016). For example, in a spatially explicit model of wetland CH_4_ fluxes from the Prairie Pothole Region, entire or portions of wetlands with dense, continuous vegetation (e.g., *Typha* spp.) were hotspots of CH_4_ emissions (Bansal et al. 2023b). However, the same lacunar air ventilation system allows for the infiltration of oxygen to the rhizosphere, which stimulates aerobic oxidation of CH_4_ through methanotrophy thereby reducing CH_4_ emission (Kao-Kniffin et al. 2010). Meanwhile, the presence of submergent aquatic macrophytes can reduce wetland CH_4_ fluxes as radial oxygen loss by the roots and can create an oxidized environment in the rhizosphere, thereby inhibiting CH_4_ production and emission (Aben et al. 2022). However, Pedersen et al. (2013) indicated that excessive build-up of submerged aquatic macrophytes in the water column inhibits light for photosynthesis and causes senescence of plant tissues, thus creating ideal conditions for high CH_4_ emissions. Future changes in wetland vegetation, whether from invasive species (Bansal et al. 2019), or from warmer temperatures (Aben et al. 2022), will undoubtedly impact wetland carbon fluxes.

Plants regulate the quality of carbon substrates by supplying initial carbon inputs through root decay and the release of exudates (Tang et al. 2018; Bansal et al. 2020; Davila and Bohlen 2021). Carbon substrate quality (i.e., lability) indicates how readily the plant carbon substrate can be decomposed through physiochemical and biological processes. Nitrogen (N) and phosphorus (P) are important elements for microbial growth; therefore, the ratios of C: N and C: P in organic matter are often used as proxies for carbon quality, which directly controls microbial community structure and organic carbon substrate utilization strategies (Krull et al. 2001; Mazurczyk and Brooks 2018; Tang et al. 2018; Luo et al. 2019; Hu et al. 2024). Plant detritus with high C: N and C: P ratio decomposes more slowly compared to detritus with low C: N and C: P ratios (Table 2) (Webster et al. 2014; Tang et al. 2018; Luo et al. 2019; Yi et al. 2023; Hu et al. 2024). In forested wetlands, plant detritus from woody deciduous species – which has a higher nitrogen content (i.e., low C: N ratio) – has been found to promote faster decomposition than woody evergreen species (Cornwell et al. 2008). Further, wetland soils with a low C: N ratio have been found to favor the growth of methanogenic bacteria, which could ultimately enhance wetland CH_4_ production and emission (refer to Sect. 4.1.1. “microbial properties) (Hu et al. 2024). Apart from C: N and C: P ratios, the lignin content of organic carbon has been widely applied as an indicator of the organic carbon decomposability (Yi et al. 2023; Bansal et al. 2023a). As the main allochthonous carbon input, plant materials with low lignin content undergo faster decomposition compared to those with high lignin content (Davila and Bohlen 2021; Yi et al. 2023). Davila and Bohlen (2021) indicated that decomposition rates of plant detritus with low lignin content in marsh wetlands are significantly (up to ten times) faster than plant detritus with higher lignin content in swamp wetlands. However, Melillo et al. (1989) indicated that the organic matter decomposition rate is controlled by lignin content only during the early stages of decomposition and is no longer regulated by the substrate quality as decomposition continues.

#### Microbial Properties

Microbes influence the rate of carbon substrate decomposition and CO_2_ and CH_4_ production (Luo et al. 2019; Mason et al. 2023). Fungi are important factors in regulating soil carbon cycling processes at the surface litter layer of wetland soils. For example, Mason et al. (2023) highlighted that arbuscular mycorrhizal fungi can facilitate conversion of labile carbon into recalcitrant carbon, thereby improving carbon sequestration potential. Further down the wetland soil profile, bacteria are an important factor in regulating soil carbon cycling processes as fungal abundance declines to levels that are orders of magnitude smaller than those of bacteria (Dang et al. 2019). Soil microbes influence the rate of carbon substrate through their regulation of enzymatic hydrolysis processes that breakdown plant detritus into smaller units by releasing extracellular enzymes to meet metabolic demands for carbon (Freeman et al. 2001; Gessner et al. 2010; Morrissey et al. 2014a). In waterlogged wetland soils, oxidative enzymes (i.e., phenol oxidase) are limited, resulting in the accumulation of phenolic materials, which subsequently inhibit hydrolytic enzymes (known as the enzymic latch hypothesis) (Freeman et al. 2001). Because of this, the organic carbon decomposition rate is lower in anaerobic conditions, resulting in organic carbon accumulation in wetland soils (Davidson and Janssens 2006; Kayranli et al. 2010; Mazurczyk and Brooks 2018). However, fungal community composition and extracellular enzyme activities are quickly reversible if wetland redox conditions change (Davidson and Janssens 2006; Kayranli et al. 2010; Neubauer and Megonigal 2021). Some studies suggest that soil microbes have a strong effect on GHG production (Tong et al. 2017; Li et al. 2019; Hu et al. 2024), but other studies suggest that carbon substate quality, quantity, and climate factors such as temperature have a stronger effect (Webster et al. 2014; Tang et al. 2018; Dalcin Martins et al. 2017). Contradictory findings from different studies suggest that our understanding of how specific soil microbes influence the mobilization and stabilization of carbon is limited (Were et al. 2019; Hu et al. 2024).

### Parent Material

#### Soil Aggregate Reactivity

Soil aggregates are formed by soil microbial activities that bind soil particles using their secretions and biomass, such as hyphal activity (Mason et al. 2023; Li et al. 2023). Soil aggregate reactivity is defined as the rate and duration of soil aggregate induced GHG production, which can be estimated by the aggregate reactor size and bulk soil properties (Luo et al. 2017; Wang et al. 2019a, b).

Aggregate reactor size is typically expressed by diameter (Brown et al. 2017; Wang et al. 2019a, b): macroaggregates (> 250 μm) represent a dynamic, younger fraction that is closely associated with plant decay, while microaggregates (< 250 μm) are a more static, older fraction (Kottkamp 2019). The greater mean pore size of macroaggregates allow faster oxygen and nutrient diffusion as compared to microaggregates (Brown et al. 2017; Kottkamp 2019; Li et al. 2023). Meanwhile, labile organic matter tends to accumulate in macroaggregates as compared to microaggregates (Wang et al. 2019a, b; Li et al. 2023). Taken together, macroaggregates tend to have a faster decomposition rate, resulting in shorter SOC preservation period (Brown et al. 2017; Mason et al. 2023; Li et al. 2023). A synthesis of the literature conducted by Wang et al. (2019a, b) supports this claim. Further, they found that despite a lack of consensus on how aggregate size impacts wetland carbon cycling, most studies (> 60%) suggest a positive relationship between aggregate size and CO_2_ production rate.

#### Cation Exchange Capacity

Soil types that are characterized by high cation exchange capacity (CEC) (e.g. luvisols and cambisols) are more likely to form soil aggregates (Vogel et al. 2014; Liu et al. 2020a; Dong et al. 2022), which provide physical protection to SOC thereby enhancing carbon sequestration (Krull et al. 2001; Liu et al. 2020a; Dong et al. 2022). CEC characterizes the ability of a specific soil to absorb and exchange positively charged chemical ions through its negatively charged surfaces (Stewart and Hossner 2001). CEC is influenced by soil texture; sandy soils have the lowest CEC values, whereas clay soils have the highest CEC values. Clay particles with high CEC are generally characterized by a greater surface area, which can provide extensive surfaces for organic matter adherence, thereby allowing for effective carbon sequestration (Rodrigues et al. 2023). Several studies have supported the notion that soils with high CEC promote the sequestration of SOC (Liu et al. 2020a; Dong et al. 2022). For instance, based on a random forest model, Dong et al. (2022) found that soil CEC was the most important variable in predicting SOC content in wetlands in China, with a significant positive correlation with SOC content.

#### Bulk Soil Properties

Bulk soil properties (e.g., bulk density, soil porosity, water holding capacity, and water filled-pore space) influence the environment surrounding the aggregate reactors, which regulate aggregate reactivity and ultimately control wetland carbon sequestration and GHG flux potential (Wang et al. 2019a, b). Soil bulk density accounts for variability in the abundance of organic matter (Horn and Smucker 2005; Brown et al. 2017). Brown et al. (2017) demonstrated that lower bulk densities are typically linked to higher organic matter levels in mineral soils. Fine-textured (i.e., clay) soils have lower bulk density compared to coarse-textured (i.e., sand) soils. Further, an increase in bulk density is associated with a decrease in soil porosity, therefore the porosity of fine-textured soils is higher compared to coarse-textured soils (Horn and Smucker 2005). The lower porosity of coarse-textured soils promotes air and water movement but limits their water holding capacity, generally resulting in low water-filled pore space (WFPS). In contrast, the high porosity of fine-textured soils has more water holding capacity, high WFPS, and less pore space for oxygen to infiltrate, thereby regulating microbial respiration and redox condition (Krull et al. 2001; Tangen and Bansal 2020). Accordingly, fine-textured soils (high WFPS) tend to accumulate more carbon as compared to coarse-textured soils (low WFPS) (Brown et al. 2017; Tangen and Bansal 2020). However, high WFPS favor the conditions for wetland CH_4_ production. Hondula et al. (2021) detected positive rates of CH_4_ emissions when the WFPS exceeded 70%. Gleason et al. (2009) detected negative rates of CH_4_ emissions when the WFPS approached 40% and peak CH_4_ fluxes when the WFPS exceeded 60%.

#### Soil Nutrient Status

Nutrient status of a wetland soil, through its controls on soil microbial activities, has also been used to explain variation in wetland carbon cycling rates (Kuzyakov 2010). Scarcity of nutrients in wetland soil can limit microbial growth, resulting in decreased organic matter mineralization rates (Kuzyakov 2002, 2010) (refer to Sect. 4.1.1. microbial properties). A few studies have shown that increasing nutrient loads to individual wetlands consistently enhance organic carbon mineralization rates resulting in higher CO_2_ emissions (Bulseco et al. 2019; Watson et al. 2022). For instance, Watson et al. (2022) found that wetlands with elevated nutrient loads exhibited increased belowground production and decomposition rates and larger CO_2_ emission rates compared to wetlands with lower nutrient loads. Meanwhile, larger nutrient loads to wetlands typically result in higher CH_4_ emissions as eutrophication favors the development of CH_4_-producing microbes (Beaulieu et al. 2019; Hambäck et al. 2023).

### Topography

#### Wetland Zonation

Wetland zonation has a large influence on wetland carbon cycling (Badiou et al. 2011; Creed et al. 2013; Tangen et al. 2015; Brown et al. 2017; Tangen and Bansal 2020). Generally, the highest SOC concentrations are more likely to be detected in the open-water zone. The lowest portion of wetlands (i.e., open water zone) are characterized by longer inundation periods, which favor the development and stability of anaerobic environmental conditions that slow decomposition rates (Richardson et al. 1994; Tangen and Bansal 2020). SOC concentrations have been found to progressively decrease from open-water zone towards the upland areas by approximately 1.5-2.0 times (Bedard-Haughn et al. 2006; Badiou et al. 2011; Creed et al. 2013; Tangen and Bansal 2020). Further, relatively fine-textured materials such as clays, silts, and loams are more likely to be found in the central and deeper portions of wetlands (i.e., open-water zone), benefiting organic carbon preservation (Tangen and Bansal 2020) (refer to Sect. 4.2.3. bulk soil properties). The high productivity of wetland vegetation (e.g., *Typha* spp.*)* in the emergent vegetation zone can promote CO_2_ uptake via enhanced photosynthesis and provide considerable AGB (Bansal et al. 2019) (refer to Sect. 4.1.1. plant community). Meanwhile, the accumulation of eroding soils in the riparian zone coupled with high productivity of wetland vegetation in the riparian zone mean that the riparian fringe around wetlands may be an area of high carbon sequestration that may be missed in estimates of wetlands as NCS (Zarrinabadi et al. 2023). Additionally, as apparent wetland boundaries can shift significantly across annual and decadal time scales, the location of areas with high carbon sequestration can move closer or farther from the wetland center, possibly storing high amounts of carbon in locations that appear unfavorable under current conditions (Bansal et al. 2016; Tangen and Bansal 2019).

### Climate

#### Climate Variability

Studies have shown that the global average temperature has increased by about 1 °C above preindustrial values because of elevated GHG concentrations in the atmosphere (Schurer et al. 2017; Baker et al. 2018). A changed climate can cause hydrological intensification (i.e., wet areas are likely to get wetter and dry areas drier, and wet periods are likely to get wetter and dry periods drier), increasing the frequency and intensity of both flood and drought events (Huntington 2006; Creed et al. 2015; Senar et al. 2018). The alteration in temperature and precipitation patterns could alter the water balance of wetland ecosystems and substantially influence wetland carbon cycling (Rezanezhad et al. 2020).

Climate-induced hydrological intensification can cause long-lasting extremes in hydrological conditions (Huntington 2006), which could alter the carbon sequestration potential (Senar et al. 2018). For instance, prolonged drought periods can result in enhanced evapotranspiration and associated water-level drawdowns, causing the exposure of top layers of the wetland to the oxygen that promotes carbon mineralization and CO_2_ production (refer to Sect. 4.4.3. water availability and 4.4.4. oxygen concentration and redox potential) (Badiou et al. 2011; Pugh et al. 2018). Meanwhile, intense flood periods can result in enhanced water and sediment in runoff, causing an increase in the supply of carbon substrates to (and from) the wetland (refer to Sect. 4.4.5. erosion and deposition).

#### Temperature

Temperature plays a vital role in shaping SOC decomposition and GHG production rates by regulating the kinetics of carbon-sensitive processes within wetlands (Davidson and Janssens 2006; Conant et al. 2011; Pugh et al. 2018; Hu et al. 2024). Increased temperature has been reported to suppress carbon sequestration rates, given the decomposition of SOM doubles with every 10 °C increase (i.e., the Q_10_ function) (Davidson and Janssens 2006), creating a positive feedback loop (i.e., increased temperature enhances the rate of organic carbon decomposition) (Kayranli et al. 2010; Moseman-Valtierra 2012; Chen et al. 2021; Hu et al. 2024). More specifically, as temperature increases, wetland carbon cycling rates typically accelerate due to amplified soil microbial activity and enzymatic reactions, resulting in enhanced breakdown of SOC compounds and production rate of gaseous end products (i.e., CO_2_ and CH_4_) (Davidson and Janssens 2006; Conant et al. 2011; Liu et al. 2021).

Although it is well-accepted that organic carbon decomposition rates are highly subject to temperature change, there is still an ongoing debate regarding whether climate change-induced increases in temperature have positive or negative effects on wetland carbon sequestration (Wang et al. 2024). Increased temperatures may cause a shift in plant phenology, which may affect plant productivity and metabolism processes (Baldwin et al. 2014; Pugh et al. 2018) (refer to Sect. 4.1.1. plant community). If the rise in plant-derived allochthonous carbon inputs to soils surpass increases in decomposition, increased temperatures will enhance wetland carbon sequestration rates (Davidson and Janssens 2006).

Temperature sensitivity (i.e., Q_10_ function) of wetland carbon kinetics depend on several other factors (e.g., carbon substrate quality, soil aggregate reactivity, and soil microbial community), imposing uncertainties in the functional relationship between temperature and wetland SOC decomposition and GHG production rates (Davidson and Janssens 2006; Liang et al. 2023; Hu et al. 2024). For example, low-quality carbon substrates are generally characterized by high activation energy of the decomposition and has been found to have higher temperature sensitivity compared to labile SOC (Kayranli et al. 2010). Further, a recent study has reported that carbon that is physically protected by soil aggregates is relative insensitive to temperature change compared to unprotected carbon, suggesting that soil aggregate-induced physical protection might be the main factor controlling the decomposition of protected carbon (Liang et al. 2023) (refer to Sect. 4.2.1. soil aggregate reactivity). Also, the influence of an increase of temperature on decomposition of SOM is dependent on the composition of SOM. Labile organic carbon is more sensitive to mineralization at higher temperatures compared to refractory organic carbon. Therefore, as temperature increases, higher levels of more recalcitrant organic carbon remain after initial stages of decomposition, resulting in slower decomposition rates (Kayranli et al. 2010) (refer to Sect. 4.1.1. plant community). While Giardina and Ryan (2000) concluded from a study of mineral clay soils that increased temperatures alone cannot increase the carbon decomposition rate, Davidson et al. (2000) challenged this conclusion, arguing that organic carbon does not exist as a single homogeneous pool (Giardina and Ryan 2000) and that the positive feedback of increasing temperature on carbon decomposition rate remained a possibility.

Temperature directly regulates CH_4_ production rate given its direct positive effect on methanogenic kinetics (Moseman-Valtierra 2012; Tangen et al. 2015; Bansal et al. 2018; Pugh et al. 2018). Tangen et al. (2015) detected a positive relationship between temperature and CH_4_ fluxes in a ponded portion of wetland catchments, with the smallest and largest CH_4_ fluxes recorded at approximately 5 °C and 15 °C, respectively. Recent studies (Chen et al. 2021; Hu et al. 2024) emphasized that CH_4_ emissions from wetlands appear to be more sensitive to changes in temperature compared to CO_2_ emissions, given the differences in the biochemical kinetics associated with methanogenesis and respiration.

Temperature can also have indirect effects on CH_4_ production rate. For example, temperature influences plant and microbial community composition and plant phenology, thereby altering carbon substrate for methanogens and regulating methanogenesis process (Kayranli et al. 2010; Bansal et al. 2016, 2020). A meta-analysis of temperature-CH_4_ relationships from globally distributed eddy covariance towers showed how the relationship can change during the growing season owing to changes in vegetation (Chang et al. 2021). Further, temperature causes enhanced evapotranspiration rates, resulting in decreased water table levels and shorter inundation periods. Therefore, the effect of changes in temperature on CH_4_ fluxes should not be viewed in isolation from the effect of changes in temperature on the wetland’s hydrological regime (Bansal et al. 2019; Chen et al. 2021). Bansal et al. (2018) found that the response of CH_4_ flux to temperature was a function of wetland inundation state. Under inundated states, thermal stimulation of the methanogenesis process results in a positive effect of temperature on CH_4_ flux rates, but under non-inundated states, microbial methanotrophy (i.e., methane oxidation) processes were found to be dominant, resulting in a negative effect of temperature on CH_4_ flux rates.

#### Water Availability

Wetland water availability, indicated by mean water table depth and the length of inundation period (or hydroperiod), affects redox potential and therefore wetland carbon storage potential (Bridgham et al. 2006; Nahlik and Fennessy 2016; Tangen and Bansal 2019; Were et al. 2019; Davila and Bohlen 2021). Prolonged inundation reduces oxygen concentrations in wetland soils, which leads to significant amounts of carbon accumulation in the surface horizon of flooded mineral soils (Neubauer et al. 2013; Lacroix et al. 2019) (refer to Sect. 4.4.4. oxygen concentration and redox potential). For instance, Davila and Bohlen (2021) found that higher mean water depth and longer mean inundation period result in higher soil carbon stocks in freshwater marshes, with mean water table depth being a stronger predictor of carbon stock than inundation period.

Wetland water availability also affects CO_2_ and CH_4_ fluxes (Gleason et al. 2009; Bansal et al. 2016; Pugh et al. 2018; Zou et al. 2022). Wetland inundation has been found to inhibit wetland CO_2_ fluxes by constraining decomposition activities of soil microbes and plant autotrophic respiration (Gleason et al. 2009; Zhao et al. 2019) (refer to Sect. 4.1.2. microbial properties). Daniel et al. (2019) and Tangen and Bansal (2020) detected the highest mean CO_2_ fluxes in drier areas near the playa wetland edge in the High Plains of the USA, which was likely due to microbial respiration being constrained by soil moisture in wetter areas. Phillips and Beeri (2008) detected the highest CO_2_ fluxes from the deep marsh zone of wetlands when there was a decrease in the soil water content, indicating significant effect of wetland hydrological condition on CO_2_ flux. Meanwhile, wetland inundation induced anoxic environmental conditions can alter plant physiological characteristics and restrain plant autotrophic respiration, ultimately inhibiting wetland CO_2_ emission (Zhao et al. 2019) (refer to Sect. 4.1.1. plant community).

The conventional relationship between wetland water table depth and CH_4_ fluxes was established based on the separation of anoxic zones (below the water table) and oxic zones (above the water table), with the net CH_4_ production occurring in the anoxic zone and CH_4_ consumption occurring in the oxic zone (Bridgham et al. 2013; Hondula et al. 2021; Ueyama et al. 2023). Zou et al. (2022) showed that CH_4_ emissions are at the lowest when the water level is well below the surface of the wetland, while CH_4_ emissions appear to be highest in flooded conditions. During inundated conditions, wetland soil is characterized by a high WFPS, which has been linked to enhanced wetland CH_4_ production and emissions (Tangen et al. 2015; Hondula et al. 2021; Bansal et al. 2023b) (refer to Sect. 4.2.3. bulk soil properties). As a result, minimal CH_4_ fluxes were generally found in upland areas, while wetland open water and emergent vegetation zone was characterized by much greater CH_4_ fluxes. However, simply understanding CH_4_ production mechanisms based on separation of anoxic zones and oxic zones has received criticism and may not comprehensively represent how wetland hydrological regimes mediate wetland CH_4_ production and release processes (Angle et al. 2017; Were et al. 2019). For instance, Angle et al. (2017) found that CH_4_ production was up to ten times higher, and methanogenesis activity was nine times greater in oxygenated soils. Their findings challenge a widely accepted assumption regarding wetland CH_4_ production and consumption. Additionally, Hondula et al. (2021) found much of the variability in CH_4_ fluxes from mineral soil wetlands was explained by direction of water level change rather than the depth of water level, indicating the importance of water level fluctuation in terms of regulating wetland CH_4_ fluxes. A CH_4_ flux study using globally distributed wetlands sites showed that pulses of CH_4_ were more common when water levels were dropping and close to the sediment surface, while CH_4_ fluxes were lower following subsequent rises in water table depth (Knox et al. 2021). Finally, despite wetlands having relatively shallow waters (typically < 2 m), water column thermal stratification and mixing can still occur due to the collected effect of surface runoff, wind, and vegetation community composition (Holgerson et al. 2022; Ahmed et al. 2023), which may impact wetland GHG production and emission. For instance, water column thermal stratification can lead to greater anoxia in the sediments, promoting CH_4_ production (Ahmed et al. 2023) (refer to Sect. 4.4.4. oxygen concentration and redox potential).

Many wetlands are subject to fluctuations between wet and dry conditions during the growing season (Tangen and Bansal 2019; Shahariar et al. 2021), leading to rapid change in oxygen concentrations and therefore their redox potential (refer to Sect. 4.4.4. oxygen concentration and redox potential). Studies have reported weak relationships between hydrological transitions (between wet and dry or vice versa) and CO_2_ fluxes; rather, the depth of water level and inundation duration are the dominant hydrological factor regulating CO_2_ flux (Tangen and Bansal 2019; Zhao et al. 2019). For instance, Tangen and Bansal (2019) detected similar CO_2_ fluxes between a “dry to wet” state and a “wet to wet” state, demonstrating current conditions have a larger influence on CO_2_ respiration compared to antecedent conditions. However, this pattern may be biased given the sampling interval (biweekly) may not have sufficiently revealed the quick response of CO_2_ fluxes to water level fluctuations (Tangen and Bansal 2019).

In contrast, studies have reported strong relationships between hydrological transitions and CH_4_ fluxes, and thus intermittently inundated wetlands are considered hotspots of CH_4_ production and emission (Badiou et al. 2011; Holgerson and Raymond 2016; Tangen and Bansal 2019; Hondula et al. 2021). Further, lower CH_4_ fluxes have been observed during rising versus falling water levels (Tangen and Bansal 2019; Knox et al. 2021), indicating that water table fluctuation may have legacy effects on CH_4_ production and emission (Pugh et al. 2018). For instance, Tangen and Bansal (2019) reported that the fluxes of CH_4_ were two times lower in the “dry to wet” state as compared to “wet to dry” state over a relatively short time scale (2 weeks). Hondula et al. (2021) supported this finding by reporting that the variability of CH_4_ fluxes was best explained by the water level change direction in the preceding week. A similar finding from Knox et al. (2021) showed CH_4_ emissions lagged changes in water table depth by a median of 17 days. This potential legacy effect has been attributed to the availability of oxidized alternative electron acceptors (Tangen and Bansal 2019; Hondula et al. 2021; Knox et al. 2021) (refer to Sect. 4.4.4. oxygen concentration and redox potential). Pugh et al. (2018) reported that the fluctuations of water table have limited impacts on wetland net carbon fluxes over multi-year timescales as compared to interannual temperature variation, indicating that the relationship between CH_4_ fluxes and water level fluctuations is sensitive to temporal scales.

#### Oxygen Concentration and Redox Potential

Wetland soil redox potential (Eh) indicates the oxidation and reduction status of the various redox couples (Table 3) (Herbert et al. 2015; Neubauer and Megonigal 2021). Given their fluctuating water levels driven by climate, wetlands provide a variety of redox conditions (Mitsch and Gosselink 2015; Neubauer and Megonigal 2021). In wetland soils, the reduction of various oxidants occurs sequentially at corresponding soil redox potentials (Kayranli et al. 2010). In aerobic environmental conditions, oxygen is always reduced first (Eh: 400-600mV). Once oxygen is completely consumed, microbes can utilize sequential anaerobic pathways to mineralize organic carbon into CO_2_ or CH_4_, which proceed in this order: nitrate is reduced to nitrous oxide (N_2_O) and nitrogen (N_2_) (~ 250mV); oxidized manganese (Mn) compounds are reduced to Mn^2+^ (~ 225mV); ferric iron (Fe^3+^) is reduced to ferrous iron (Fe^2+^) (~ 100 mV); sulfate (SO_4_^2−^) is reduced to sulfide (S^2−^) at (~-100mV); and finally CO_2_ is reduced to CH_4_ at (~-200mV) (Baldwin et al. 2006). This sequential reduction, also known as the “redox ladder” (Borch et al. 2010), controls the residence time of carbon in the soil (Reddy and DeLaune 2008; Kayranli et al. 2010; Neubauer and Megonigal 2021). Given that wetland CH_4_ production results from microbes utilizing CO_2_ as the final electron acceptor in their metabolic pathways (Poffenbarger et al. 2011), the introduction of alternative terminal electron acceptors such as nitrate, ferric iron, and sulfate can inhibit rates of methanogenesis (Table 3) (Neubauer et al. 2013; Herbert et al. 2015). For instance, when water levels are low, higher soil redox potential allows alternate electron acceptors (e.g., Fe^3+^) to become re-oxidized, stimulating microbial competition for carbon resources, which ultimately inhibits CH_4_ production after periods of lower water levels (Hondula et al. 2021).

**Table 3:** The general effect of redox induced alternative terminal electron acceptor (alt. e^−^) transformations on wetland carbon cycle

Redox range	Terminal electron Acceptor	Redox-induced (alt. e^−^) transformations effects of carbon	References
+ 200 to + 300mV	NO_3_^−^	Under anoxic environmental condition, an increase in the concentration of NO_3_^−^ could stimulate CO_2_ production *via* enhanced organic matter mineralization. The presence of NO_3_^−^ could lead to denitrification which converts NO_3_^−^ to N_2_ using NO_3_^−^ as the alternative e^−^, resulting in reduced CH_4_ production and emission.^1^	Herbert et al. (2015)
+ 100 to + 200mV	Mn^4+^	Under anaerobic conditions, Mn oxides are strong oxidizing agents, which could facilitate microbial degradation of organic matters, leading to increased CO_2_ production. Meanwhile, the presence of Mn^4+^ could change the primary pathway of anaerobic metabolism from methanogenesis to higher energy-yielding pathways, such as Mn^4+^ reduction.^1^	Herbert et al. (2015)
-100 to + 100mV	Fe^3+^	Redox-induced Fe transformation (Fe^3+^ reduced to Fe ^2+^) reduces carbon sequestration and promotes GHG fluxes. Under anaerobic conditions, the presence of Fe^2+^ enhances activities of phenol oxidase, b-glucosidase, and hydrolytic enzymes activities, promoting organic carbon mineralization and CO_2_ production, challenging the enzyme latch hypothesis.^1^	Hall and Silver (2013)
-200 to -100mV	SO_4_^2−^	Redox-induced sulfur (S) transformation typically increases organic matter mineralization rate and reduces CH_4_ fluxes^1^. Elevated sulphate (SO_4_^2−^) concentrations typically lead to increases in SO_4_^2−^ reduction rates and the overall enhancement of organic matter mineralization in freshwater wetland soils. Under relatively oxic conditions, S occurs as oxidized forms: SO_4_^2−^, which can be reduced to sulfide (S^2−^) via anaerobic microbial metabolism, which is a higher energy-yielding pathway compared to methanogenesis. Accordingly, the presence of SO_4_^2−^could reduce CH_4_ production and emission.^2^	Herbert et al. (2015), Neubauer et al. (2013), Villa and Bernal (2018), Poffenbarger et al. (2011)
-300 to -200mV	CO_2_ reduced to CH_4_	In anoxic environmental conditions and in the absence of alternate metabolic pathways, CH_4_ is produced by methanogens using acetate or CO_2_ as electron acceptors via methanogenesis process, which is a low energy-yielding metabolic pathway that generates CH_4_ as the final product of metabolism. However, recent evidence shows that CH_4_ can be produced in the presence of SO_4_^2−^ via methylotrophic methanogenesis.	Herbert et al. (2015), Neubauer and Megonigal (2021), Baldwin et al. (2006), Poffenbarger et al. (2011), Dalcin Martins et al. (2017), Narrowe et al. (2019), Capooci et al. (2024)

^1^Salinization of freshwater wetlands is generally associated with rapid increase in the concentration of alternative terminal electron acceptors (alt. e^−^) (e.g., Fe^3+^, SO_4_^2−^, NO_3_^−^, Mn^4+^), which subsequently enhance overall organic matter mineralization and increase CO_2_ production rate (Herbert et al. 2015; Shahariar et al. 2021) but decrease CH_4_ production rate (Baldwin et al. 2006; Poffenbarger et al. 2011; Herbert et al. 2015)

^2^In instances with high dissolved organic carbon (DOC), sulphate reduction and methanogenesis can co-occur (Dalcin Martins et al. 2017)

#### Erosion and Deposition

Climate-driven wind and water movement has been found to accelerate the processes of wetland soil erosion and deposition, altering carbon storage and fluxes within wetlands (Fennessy et al. 2018). Soil loss by wind erosion is generally found to be most pronounced on the upland area in agricultural landscapes, which is driven by the predominant wind direction (Zarrinabadi et al. 2023). Meanwhile, a significant portion of sediment flux into wetlands is primarily attributed to water movement (Zarrinabadi et al. 2023). For instance, it is estimated that spring meltwaters account for 80% of the total annual runoff into the wetlands of the Canadian Prairie Pothole Region (Glozier et al. 2006). A recent study conducted in prairie pothole wetlands show that soil erosion generally detected within the upland areas, but soil deposition within the riparian and wetland areas, with most of the eroded soils deposited in the riparian area and a minority deposited in the open water area of wetlands (Zarrinabadi et al. 2023). The small portion of sediment deposited in the open water area of wetlands suggests that carbon accumulation at the center of prairie pothole wetlands is more likely from autochthonous sources (e.g., dead plant and microbial biomass). Deposition of eroded soils results in accumulation of not only carbon but nutrients, with nutrient enrichment linked to enhanced organic matter production (refer to Sect. 4.2.4. soil nutrient status) and the arrival of highly productive invasive species (e.g.,* Typha* spp.) (Fennessy et al. 2018; Bansal et al. 2019) (refer to Sect. 4.1.1. plant community), which could ultimately alter wetland CO_2_ and CH_4_ fluxes.

## Human Activity

**Fig. 5:**
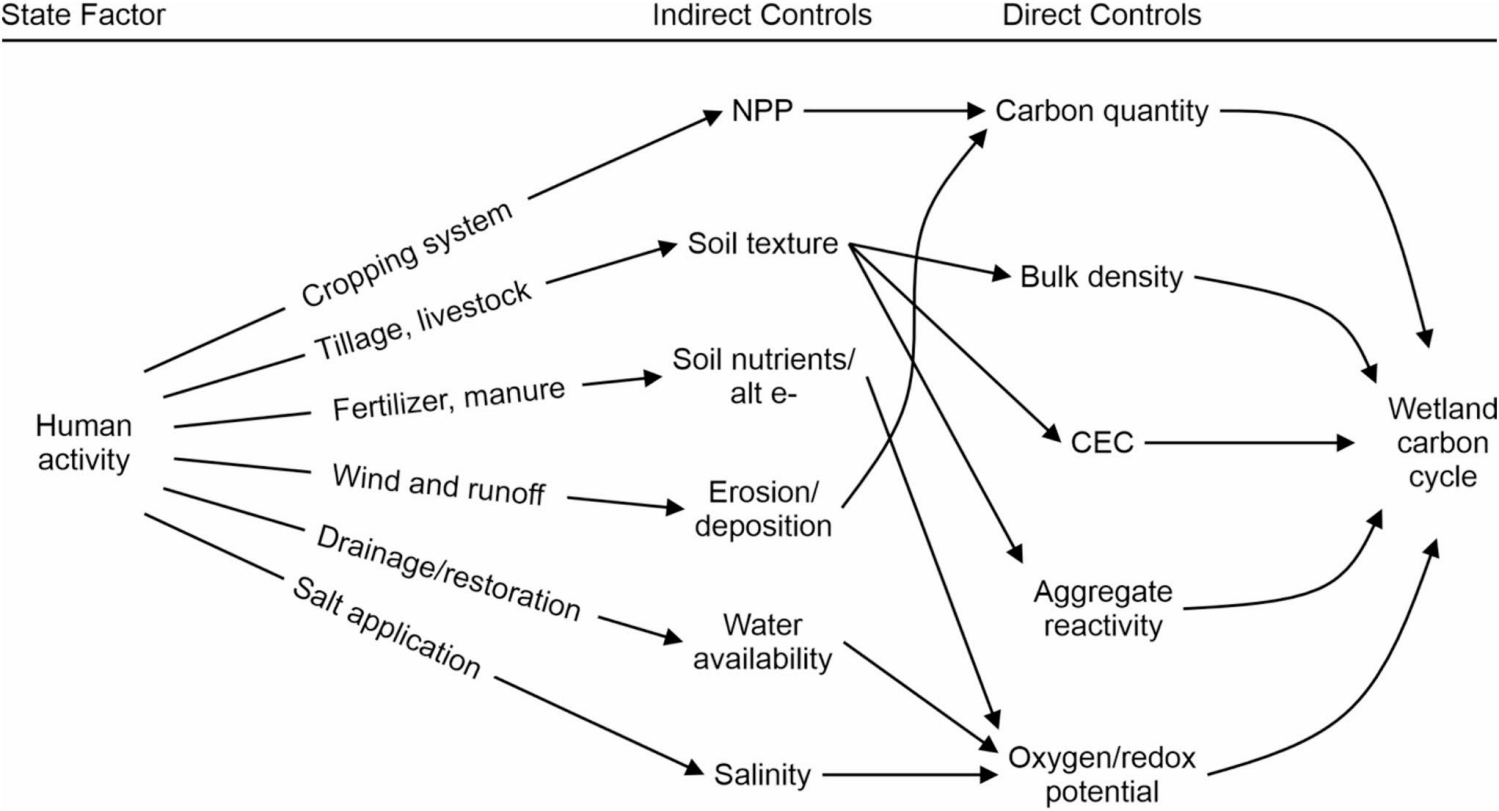
Conceptual diagram showing how different wetland management options affect wetland physical, chemical, and biological characteristics and wetland carbon cycling processes, including carbon sequestration and greenhouse gas (GHG) flux rates. C: N stands for carbon to nitrogen ratio; CEC stands for cation exchange capacity; NPP stands for net primary productivity; alt e- stands for alternative terminal electron acceptors

Human activities, combined with climate change, can significantly alter numerous indirect and direct factors that regulate the wetland carbon cycle (Fig. 5) (Bansal et al. 2016; Fennessy et al. 2018; Shahariar et al. 2021). Freshwater mineral soil wetlands are generally found in temperate climate regions where anthropogenic land use change is most pronounced, making them particularly vulnerable to human activities (Nahlik and Fennessy 2016; Loder and Finkelstein 2020). Studies have shown that disturbances to wetland ecosystems caused by human activities (e.g., road salt application, agricultural practices, etc.) can reduce the carbon sequestration potential of mineral soil wetlands, leading to increased GHG emissions and further intensifying climate change (Bridgham et al. 2006; Nahlik and Fennessy 2016; Bansal et al. 2016). The following subsections discuss how human activities influence the wetland carbon cycle by modifying both indirect and direct controlling factors.

### Freshwater Salinization

Freshwater salinization due to human activities (e.g., road salt application) will result in quick (between days and weeks) changes in the ionic concentrations and chemical equilibria (Baldwin et al. 2006; Haq et al. 2018; Kaushal et al. 2021). A negative relationship has been reported between salinity and organic matter content (Nahlik and Fennessy 2016; Luo et al. 2019; Li et al. 2019). Luo et al. (2019) found that low salinity wetlands favor high primary productivity, while more saline wetlands have a stronger carbon mineralization capability, producing and emitting more CO_2_. Neubauer et al. (2013) found a similar positive relationship between salinity and organic carbon mineralization rate. However, they detected a declining trend in soil CO_2_ production rate over time due to prolonged exposure to salinity, which suggests that the positive relationship between salinity and carbon mineralization rate may vary over time, depending on other controlling factors (e.g., the quantity and quality of carbon substrate inputs). Zhang et al. (2023) found that the salinity dependence of wetland CO_2_ emission is subject to antecedent saline conditions. Decreasing salinity inhibited CO_2_ production and emission from saline wetlands, while increasing salinity inhibited CO_2_ production and emissions in freshwater wetlands, suggesting that the specific effect of salinity on organic carbon decomposition and CO_2_ emissions may vary among different types of wetlands.

Salinization of wetlands suppresses CH_4_ production and emission rates by shifting the predominant anaerobic metabolism pathway from methanogenesis to pathways that produce higher energy yields (e.g. sulfate reduction) (Baldwin et al. 2006; Poffenbarger et al. 2011; Herbert et al. 2015) (refer to Sect. 4.4.4. oxygen concentration and redox potential). Poffenbarger et al. (2011) found that a polyhaline wetland had significantly lower CH_4_ fluxes compared to other wetlands with lower salinity level. Soued et al. (2024) similarly observed that prairie pothole wetlands characterized by elevated salinity had lower CH_4_ emissions. However, some studies have found notable exceptions to the expected effect (Dalcin Martins et al. 2017; Capooci et al. 2024). Dalcin Martins et al. (2017) found that the presence of extremely high DOC concentrations in some prairie pothole wetlands can provide ample carbon substrates to support both sulfate-reduction and methanogenesis. Recent studies explored the mechanisms and relative importance of methylotrophic methanogenesis, which produces CH_4_ in the presence of sulfate using non-competitive substrates (e.g., methanol, methylsulfides, and methylamines) (Dalcin Martins et al. 2017; Narrowe et al. 2019; Capooci et al. 2024).

The effect of salinization on wetland carbon stores and fluxes may vary due to other process controls (Baldwin et al. 2006; Poffenbarger et al. 2011; Neubauer et al. 2013; Morrissey et al. 2014b; Herbert et al. 2015). For example, some studies have suggested that increasing salinity can alter the soil microbial community structure (Neubauer et al. 2013; Morrissey et al. 2014b) and enhance certain extracellular enzyme activities (e.g., phenol oxidase), which subsequently increases the carbon mineralization rate (Morrissey et al. 2014b; Luo et al. 2019). In contrast, other studies have suggested that hypersalinity inhibits certain extracellular enzyme activity, such as phenol oxidase and glucosidase, since hypersalinity can impose adverse effects on the stability of molecular and conformational states of proteins (Neubauer et al. 2013; Li et al. 2019).

### Agricultural Activities

Wetland drainage for agricultural purposes can alter wetland water availability and redox conditions by removing excess water from the soil surfaces. Enhanced oxygen infiltration increases organic carbon oxidation, resulting in enhanced organic carbon decomposition and CO_2_ production rate and the depletion of wetlands soil organic carbon stock (Bridgham et al. 2006; Badiou et al. 2011; Brown et al. 2017).

Conventional tillage practices on drained wetlands can further cause significant loss of SOC (Bedard-Haughn et al. 2006; Badiou et al. 2011; Brown et al. 2017). Euliss et al. (2006) indicated that, compared to tilled cropland, undisturbed wetlands can store roughly twice the amount of organic carbon. Tillage regulates wetland carbon sequestration potential mainly through altering soil structures (e.g., soilaggregate and bulk density) (Bedard-Haughn et al. 2006; Brown et al. 2017; Tangen and Bansal 2020) (refer to Sect. 4.2.1. soil aggregate reactivity and Sect. 4.2.3, bulk soil properties). Further, tillage practices, along with wind and water movement, result in the net downslope movement of soils and ultimately cause soil loss over the upland area and deposition in the wetland central areas, or soil loss from convex profiles and accumulation in concave profiles, altering carbon storage and fluxes within wetlands (Li et al. 2008) (refer to Sect. 4.4.5. erosion and deposition).

Excessive fertilizer application can lead to high nutrient loading rates which can ultimately result in enhanced CH_4_ production (Beaulieu et al. 2019; Hambäck et al. 2023) as well as potentially harmful cyanobacteria blooms (Erratt et al. 2018, 2023). Nutrient loading can also alter the emergent and submerged macrophyte communities and production leading to changes in carbon sequestration rate (Short et al. 2016) (refer to Sect. 4.2.4. soil nutrient status). Additionally, input of pesticides from surrounding agricultural landscapes can impact soil microbes, plants, and invertebrates within wetlands, thereby altering carbon accumulation and GHG flux mechanisms (Beaulieu et al. 2019; Hambäck et al. 2023; Cornish et al. 2024).

Despite progress towards understanding how agricultural activities (e.g., tillage practice and fertilizer application) and wind and water erosion affect soil movement and carbon fluxes in wetlands (Fennessy et al. 2018; Zarrinabadi et al. 2023), there is lack of consensus regarding the extent soil movement has caused changes in the short-term rates of carbon accumulation in different wetland zones which limits our understanding on long-term carbon balances in wetlands (Loder and Finkelstein 2020).

## Managing Wetlands as Natural Climate Solutions

### Individual Wetlands

Understanding the mechanisms associated with how different physical, chemical, and biological factors interactively, directly, and indirectly, regulate wetland carbon sequestration potential and CO_2_ and CH_4_ fluxes is an important prerequisite for effective implementation of best management practices to promote wetlands as NCS. The ability of wetlands to act as NCS must account for both atmospheric CO_2_ uptake and wetland CO_2_ and CH_4_ emissions (Ferreira et al. 2023; Hambäck et al. 2023). Given the long atmospheric lifetime of CO_2_ and high radiative efficiency of CH_4_, effective wetland management practices should involve simultaneous but separate efforts to enhance wetland carbon sequestration potential and reduce GHGs emitted from wetlands. Here, based on understanding of the state and anthropogenic factors influencing wetland carbon cycling, we present a comprehensive framework of best management practices that policymakers and practitioners can use to enhance the wetland carbon sink potential by offering four categories of wetland management strategies – protect, rewet, avoid, amend.

**Protect**: Protection of intact wetlands and their surrounding riparian buffers is beneficial because wetlands have the greatest potential to sequester carbon and reduce CO_2_ and CH_4_ production and emission (Tangen and Bansal 2020; Creed et al. 2022; Zou et al. 2022). Prioritization could be based on wetland size and shape as these characteristics are among the most accessible and observable features available to practitioners through ground surveys or satellite imagery. For example, small wetlands (< 1 ha) with large perimeter: area ratios and shallow water depths have a greater potential to support robust, emergent vegetation, which benefits wetland carbon sequestration (Marton et al. 2015; Van meter and Basu 2015). Wetlands that are relatively small tend to have shorter hydroperiods and drier soils than larger wetlands, reducing the period of CH_4_ production and enhancing the potential for CH_4_ oxidation (Bansal et al. 2023b), but also increasing the period of CO_2_ emissions. Large wetlands (> 10 ha) with higher groundwater inputs can have increased salinity levels that limit methanogenesis processes, resulting in relatively lower CH_4_ fluxes. Medium sized wetlands (i.e., 2–4 ha) are in the “goldilocks” zone of producing and emitting CH_4_ given their longer hydroperiod as compared to smaller wetlands (< 1 ha) and lower likelihood of saline levels that limit methanogenesis (Bansal et al. 2023b). Further, wetland morphology must be taken into account when prioritizing protection of intact wetlands – e.g., wetlands with a large wetland area: catchment area ratio may increase carbon sequestration by enhancing the capacity of the wetland to store water and retain nutrients (Hambäck et al. 2023); wetlands with a large perimeter to area ratio may increase the proportion of emergent vegetation contributing to carbon sequestration potential (Van meter and Basu 2015); and wetlands with concave profiles have more frequent wetting and drying cycles as compared to those with convex profiles (Marton et al. 2015), resulting in relatively little change in CO_2_ production but enhanced CH_4_ production (Creed et al. 2013) (refer to Sect. 4.4.5. oxygen concentration and redox potential). Finally, wetlands that are persistently inundated resulting in persistent anaerobic conditions can result in a larger wetland carbon sequestration potential (Creed et al. 2022).

**Rewet**: Rewetting drained wetlands by raising the water table to the level prior to drainage (IPCC 2014) will reintroduce anaerobic environmental conditions, thereby reducing SOC mineralization rates (Davidson and Janssens 2006; Kayranli et al. 2010; Mazurczyk and Brooks 2018). While rewetting drained wetlands can slow the rate of SOC loss, the recovery time of SOC stocks may vary depending on other factors such as vegetation characteristics and land use type in upland areas (Bedard-Haughn et al. 2006; Tangen and Bansal 2020; Neubauer and Megonigal 2021). Further, the magnitude of change in CO_2_ fluxes following wetland rewetting is subject to various factors including, but not limited to climate, antecedent water level and soil chemical composition (Neubauer and Megonigal 2021). When rewetting drained wetlands, efforts should be made to maintain stable water levels in the rewetted wetlands, since fluctuating water levels have been linked to enhanced wetland CH_4_ production and emission rates (Badiou et al. 2011; Holgerson and Raymond 2016; Tangen and Bansal 2019; Hondula et al. 2021; Zou et al. 2022).

**Avoid**: Avoiding certain agricultural practices can minimize carbon mineralization and reduce CO_2_ and CH_4_ fluxes. First, fertilizer application on cultivated drained wetlands can be detrimental as it can increase nutrient (i.e., nitrogen and phosphorus) loading in the drained wetlands, thereby enhancing leaf and root litter decomposition, and ultimately increasing CO_2_ fluxes (Brown et al. 2017). Second, excess tillage in surrounding or in cultivated drained wetland areas can be detrimental as it can enhance soil aeration, erosion and reduce soil aggregation, resulting in increased decomposition of SOC due to the loss of physical protection offered by soil aggregation under relatively-oxic condition (Bedard-Haughn et al. 2006; Brown et al. 2017; Mason et al. 2023). Meanwhile, excessive tillage practice has been found to reduce certain soil microbial activity (e.g., arbuscular mycorrhizal fungi), which further causes increased SOC decomposition rate (Mason et al. 2023). Third, the application of heavy equipment on cultivated drained wetlands can have negative effects. Cultivated areas always have the highest soil bulk densities due to compaction caused by heavy equipment, resulting in dramatic changes in belowground properties and processes (Tangen and Bansal 2020). Fourth, animal grazing in wetlands embedded in or adjacent to croplands can be detrimental unless conducted in a sustainable manner. Grazing can reduce carbon sequestration by trampling vegetation, enhancing soil compaction, and increasing GHG emissions from nutrient-rich manure inputs (Limpert et al. 2021; Finocchiaro et al. 2014). Yet, light grazing can positively control the overgrowth of plants, which has been linked to reduced above-ground biomass and lower CO_2_ emissions (Finocchiaro et al. 2014), enhancing the efficacy of wetlands as NCS. Finally, the application of road salts can harm or benefits wetlands. While, saline wetlands can enhance SOC mineralization and CO_2_ production rate, there is some evidence of an optimal salinity level that maintains SOC mineralization rates and CO_2_ production rate but inhibits CH_4_ production (Herbert et al. 2015; Shahariar et al. 2021).

**Amend**: Wetland managers can apply amendments to intact and restored wetlands to maximize carbon sequestration and mitigate CO_2_ and CH_4_ production. Some innovative ideas to increase wetland carbon sequestration potential and decrease CO_2_ and CH_4_ production and emissions include the following. First, apply soil amendments such as biochar and sulphate to reduce GHG emissions given their ability to modify soil microbial community composition and increase soil water holding capacity and form soil aggregates (Han et al. 2016; Mason et al. 2023; Mosa et al. 2023). However, given that artificial amendments may have unintended consequences, their application should be scientifically-based, with appropriate monitoring for their potential positive and negative effects before being broadly adopted (Ritson et al. 2021; Mason et al. 2023). Second, modify the microbial community composition to enhance the abundance of arbuscular mycorrhizal fungi that could facilitate the conversion of labile carbon to recalcitrant carbon and thus increase the carbon sequestration potential (Mason et al. 2023), or to enhance sulphate-reducing bacteria that could inhibit CH_4_ production and emission in wetlands (Mason et al. 2023). However, manipulating the soil microbial community to influence wetland carbon cycling is a complex process and requires interdisciplinary research collaboration (Ritson et al. 2021). Third, modify the plant community composition in uplands to plants with a high C: N ratio that inhibit CO_2_ and CH_4_ fluxes (Schultz and Pett 2018), in riparian areas to plants with high lignin contents to increase carbon sequestration potential (Villa et al. 2020), and in wetlands to cut, graze, or crush *Typha* to reduce CH_4_ fluxes (Bansal et al. 2019; Johnson et al. 2021). It should be noted that the functional relationship between riparian plant composition and wetland carbon fluxes is subject to many factors such as the density, species, and age of plants in addition to the lignin content (Udawatta and Jose 2012; Cole et al. 2020). For example, riparian buffers dominated by woody species could stabilize the wetland slope, thereby reducing soil erosion from uplands entering the wetlands (Cole et al. 2020; Zarrinabadi et al. 2023), and can enhance soil complexity, porosity, and permeability, which increase soil infiltration and water holding capacities (Tufekcioglu et al. 2003; Cole et al. 2020). Accordingly, wooded riparian buffers can be highly efficient in sequestering carbon (Tufekcioglu et al. 2003; Udawatta and Jose 2012). Finally, the riparian buffer width surrounding wetlands should be optimized to restrict carbon and nutrients from erosion (Creed et al. 2008) and runoff from entering the wetland (Cole et al. 2020; Pasut et al. 2021), thereby reducing wetland CO_2_ and CH_4_ fluxes (Bridgham et al. 2013). Wider riparian buffer widths are more effective at trapping sediments and nutrients given the increased distance from agricultural practice and the greater area of riparian vegetation to intercept the sediments (Cole et al. 2020). However, excessive riparian buffer width, beyond what is essential, is economically impractical and may lead to resistance to adoption (Buckley et al. 2012).

Despite offering several options to manage wetlands as an effective NCS, it should be acknowledged that some management practices may simultaneously benefit wetland carbon sequestration and GHG fluxes, limiting their efficacy in terms of promoting wetlands as net carbon sinks. For instance, controls such as substrate quantity (higher), wetland size (small size with complex shorelines) and topography (steep slope) favor both carbon sequestration and CH_4_ emission  . Additional research is therefore needed to understand the functional relationships between controlling factors and wetland carbon cycling and, thereby, predict their influence on carbon sequestration and CH_4_ emission. Meanwhile, some of the direct controls of carbon sequestration and GHG fluxes remain poorly understood or have complex relationships that are difficult to tease apart (Tangen and Bansal 2020; Davila and Bohlen 2021). For instance, it remains unclear if specific salinity or sulfate concentrations can achieve a balance between methanogenesis and methanotrophy, thereby allowing wetland CH_4_ emissions to become negligible in the context of wetlands as NCS (Poffenbarger et al. 2011; Herbert et al. 2015; Luo et al. 2019; Zhang et al. 2023).

### Wetlandscapes

Wetland carbon sequestration and GHG flux mechanisms depend not only on the properties of individual wetlands, but also on the number and distribution of individual wetlands (Marton et al. 2015; Lee et al. 2023) (Fig. 1). As many studies have noted (Cohen et al. 2016; Mushet et al. 2015; Ameli and Creed 2017), hydrologically connected depressional wetlands release water across the landscape through both surface water fill-and-spill mechanisms and subsurface pathways (Cohen et al. 2016; Ameli and Creed 2017; Mushet et al. 2015; Lee et al. 2023). The prevalence of hydrological connections via either surface or subsurface pathways is regulated by short-term weather and long-term climate conditions (Euliss et al. 2004; Cohen et al. 2016; Lee et al. 2023). During wet stagnant conditions (i.e., in the absence of flowing water), wetlandscape connectivity is established solely through subsurface pathways. In contrast, during wet flowing conditions, surface connectivity pathways become activate due to the water stored in the wetland exceeding the threshold for basin overflow (Lee et al. 2023).

Management options that can enhance carbon sequestration potential and reduce CO_2_ and CH_4_ emissions from wetlandscapes include the following. First, it is important to recognize that wetlands are not isolated entities but form an integral component of the wetlandscape (Rains et al. 2016; Thorslund et al. 2017). The diversity of physical, chemical, and biological characteristics is important for hydrological and ecological resilience of the wetlandscape and the wetland functions and services they support (Cohen et al. 2016; Creed et al. 2017; Thorslund et al. 2017; Lane et al. 2023). Therefore, wetlands ranging from relatively hydrologically connected to disconnected should be targeted in the design of wetlands as NCS, with each wetland configured to optimize its role as a climate solution within the wetlandscape. Specific ways to manage wetlandscapes will vary, depending on their context, but will likely require interdisciplinary research that involves a combination of field-based measurements, computer-based geographic information systems, remote sensing, and numerical or statistical modeling techniques (Thorslund et al. 2017). Second, hydrological drainage of individual wetlands within the wetlandscape should be carefully considered and designed, since the draining of individual wetlands for agricultural purposes has been found to cause increased nutrient loading to enter downstream waterbodies (i.e., wetlands, streams, rivers, and lakes), which may cause severe environmental, economic, and social consequences (Marton et al. 2015; Stewart 2015). Third, in terms of the delivery of wetland ecosystem services, it is important to consider the potential for synergies (i.e., the increase of one service causes an increase in another service) and tensions (i.e., the increase of one service causes the decrease in another service) (Zamberletti et al. 2018; Ferreira et al. 2023). For instance, an increase in nutrient retention within a wetland may hinder biodiversity, given that excessive nutrients can lead to a less biodiverse wetland plant community (Zamberletti et al. 2018; Hambäck et al. 2023).

## Conclusion

There is an increasing focus on utilizing wetlands as NCS to support nations in achieving and enhancing their commitments to net-zero GHG emissions by 2050. Wetland conservation (avoided conversion) and restoration are considered effective means of promoting wetlands as NCS. While wetlands can remove atmospheric CO_2_ through carbon sequestration, they are also considered one of the largest natural CH_4_ sources. The role of wetlands as either carbon sinks or sources depends on a suite of physical, chemical, and biological factors that indirectly and directly regulate the ratio of CO_2_ sinks to CH_4_ sources. One must consider the interactive effects of process controls on wetland carbon cycling when estimating the carbon sequestration rates and CO_2_ and CH_4_ flux rates. Further, one must design management interventions that can increase carbon sequestration potential while reducing GHG emissions. Managing both individual wetlands and wetlandscapes is crucial for achieving the full range of benefits that wetlands can provide, including but not limited to climate change mitigation.

## Data Availability

This review did not include data collection.
